# Improved pig behavior analysis by optimizing window sizes for individual behaviors on acceleration and angular velocity data

**DOI:** 10.1093/jas/skac293

**Published:** 2022-09-03

**Authors:** Saleh Alghamdi, Zhuqing Zhao, Dong S Ha, Gota Morota, Sook S Ha

**Affiliations:** The Bradley Department of Electrical Engineering, Virginia Polytechnic Institute and State University, Blacksburg, VA 24061, USA; The Bradley Department of Electrical Engineering, Virginia Polytechnic Institute and State University, Blacksburg, VA 24061, USA; The Bradley Department of Electrical Engineering, Virginia Polytechnic Institute and State University, Blacksburg, VA 24061, USA; School of Animal Sciences, Virginia Polytechnic Institute and State University, Blacksburg, VA 24061, USA; The Bradley Department of Electrical Engineering, Virginia Polytechnic Institute and State University, Blacksburg, VA 24061, USA

**Keywords:** data segmentation, labeling, pig behavior classification, pig behavior monitoring, window size, wireless sensor node

## Abstract

This paper presents the application of machine learning algorithms to identify pigs’ behaviors from data collected using the wireless sensor nodes mounted on pigs. The sensor node attached to a pig’s back senses the acceleration and angular velocity in three axes, and the sensed data are transmitted to a host computer wirelessly. Two video cameras, one attached to the ceiling of the pigpen and the other one to a fence, provided ground truth for data annotations. The data were collected from pigs for 131 h over 2 mo. As the typical behavior period depends on the behavior type, we segmented the acceleration data with different window sizes (**WS**) and step sizes (**SS**), and tested how the classification performance of different activities varied with different WS and SS. After exploring the possible combinations, we selected the optimum WS and SS. To compare performance, we used five machine learning algorithms, specifically support vector machine, *k*-nearest neighbors, decision trees, naive Bayes, and random forest (**RF**). Among the five algorithms, RF achieved the highest F1 score for four major behaviors consisting of 92.36% in total. The F1 scores of the algorithm were 0.98 for “eating,” 0.99 for “lying,” 0.93 for “walking,” and 0.91 for “standing” behaviors. The optimal WS was 7 s for “eating” and “lying,” and 3 s for “walking” and “standing.” The proposed work demonstrates that, based on the length of behavior, the adaptive window and step sizes increase the classification performance.

## Teaser

Our contribution is using the adaptive window and step sizes to classify behaviors with different durations so that the windows could precisely capture the duration of various behaviors and increase the number of segments to alleviate the skewed class distribution. The proposed work demonstrates that the optimal window and step sizes based on the length of behavior increased the classification performances.

## Introduction

Providing proper animal care is vital for maximizing productivity and ensuring animal welfare. However, current labor-based monitoring practices fall short in providing quality care for the animals because the assessments of animal health are often subjective. Sensor-based animal monitoring technology can provide objective assessments of animal health and continuous care of animals in comparison to a glance at an animal’s status by animal care personnel. In addition, the application of machine learning-based decision-supporting tools can lift the burden of analyzing the immense amount of data that is generated by the sensor system (Nardone et al., 2004).

Several studies demonstrate that wireless sensor nodes (**WSN**) integrated with machine learning can monitor pigs and identify their behaviors (Cornou and Lundbye-Christensen, 2008, 2010; Escalante et al., 2013; Thompson et al., 2016; Haladjian et al., 2017). Sensors for WSN can include an accelerometer, gyroscope, magnetometer, heartbeat rate sensor, and temperature sensor. Typical pig behaviors we considered for the classifications are “walking,” “eating,” “drinking,” “standing,” “sitting,” and “lying.” Several machine learning algorithms used for the classifications of pigs’ behaviors include support vector machine (**SVM**) (Thompson et al., 2016), LogitBoost (Escalante et al., 2013), and *k*-nearest neighbors (**KNN**) (Haladjian et al., 2017). One study (Haladjian et al., 2017) measured acceleration on pigs and compared the performance of those three machine learning algorithms to classify three behaviors, “walking,” “eating,” and “resting.” The KNN algorithm achieved the maximum average precision of 80.04%. However, they collected data over a short period of less than an hour from only one pig, leading to possibly a biased result.

Another study for pigs’ behavior identification, conducted by Thompson et al. (2016), used SVM to classify three variations of “lying” postures (lying on the belly, lying on the right side, lying on the left side), “standing,” and “sitting.” They identified transitions between postures, such as the transitions between lying on the belly and lying on the side. As the study aimed to protect piglets from getting crushed by sows during parturition, transition detection was helpful. The highest F1 score was 0.77 across all classes. The classifier could not distinguish some activities, such as “standing” and “sitting,” and did not identify “eating” and “walking” behaviors.

A third (Escalante et al., 2013) classified pigs’ behaviors, specifically “feeding,” “rooting,” “walking,” “sternal lying,” and “lateral lying,” using the same data set described in Cornou and Lundby-Christensen (2008, 2010). A LogiBoost classifier correctly classified by a factor of 74.64% on the average under the observations with 1 s. However, the accuracy increased to 80% with longer observations of 2 min. The data were collected for only 30 min for each behavior, which might not have been long enough for a robust data set.

Most existing studies for the classifications of pigs’ behaviors have one or a combination of the following shortcomings (Cornou and Lundbye-Christensen, 2008; Escalante et al., 2013; Thompson et al., 2016). First, the data collection period is short, which might be insufficient for generalization. Second, the classification performances leave room for improvement due to noisy labeling or imbalanced class distribution. Third, and most important, previous studies used predetermined window sizes (**WS**) and step sizes (**SS**) and treated all behaviors equally to compute features. However, from our observation, the pigs have various behavior patterns with different durations. Thus, treating behaviors with a small duration the same as those with a long duration could inaccurately label multiple different behaviors within the window frame as a single behavior, and the less frequent behaviors in the window frame might be ignored.

**Figure 1.**
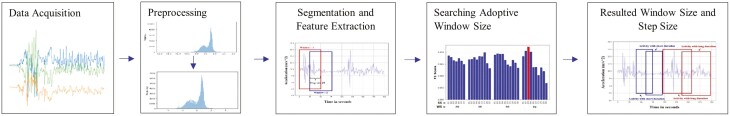
The General Workflow: Data Acquisition to collect data, Preprocessing to clean motion data, Segmentation and Feature Extraction to prepare for classification, Searching Adaptive Window Size using the classification, and Customizing the Window Size and Step Size for individual activities with adaptive window.

This study aimed to improve the classification performances to identify pigs’ behaviors. The general workflow is summarized as follow. Data acquisition to collect data, preprocessing to clean and standardize motion data, segmentation, and feature extraction to prepare for classification, searching adaptive WS and SS based on the classification results, and customizing adaptive WS and SS for individual activities (Figure 1).

Our contribution is summarized as follows: We 1) built a WSN capable of measuring acceleration and angular velocity accurately, and 2) collected data for 131 h over 2 mo from pigs of varying sizes. Using videos taken from two cameras, 3) manually labeled behaviors of the pigs into “walking,” “eating,” “standing,” “lying,” “sitting,” “drinking,” “playing,” and “unknown” to avoid any mechanical errors. We 4) compared five machine learning algorithms to identify pigs’ behaviors, including “walking,” “eating,” “lying,” and “standing.” Finally, we 5) identified the optimal WS and SS via the adaptive window method by investigating the classification performances of individual behaviors and used the combined optimal WS and SS to characterize the four behaviors.

## Materials and Methods

### Animal procedures

All animal experiments were approved and carried out in accordance with the Virginia Tech Institutional Animal Care and Use Committee.

### Sensors

**Figure 2.**
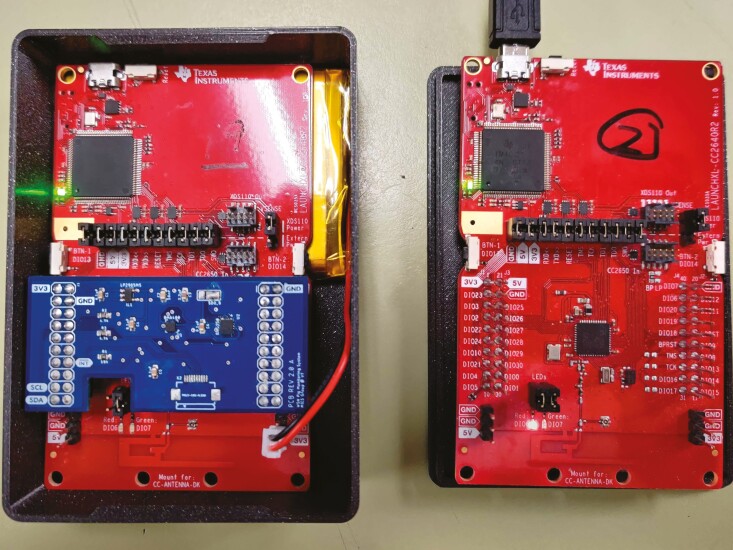
Our wireless sensor nodes (WSNs) used to collect acceleration and angular velocity data.

The WSN was prototyped using a Texas Instruments CC2640R2F microcontroller with an embedded Bluetooth low-energy radio for wireless data transmission (Texas Instruments, 2021). To measure acceleration and angular velocity, the WSN has two sensors, a 3-axis accelerometer BMA400, and a 3-axis gyroscope BMG250. Both sensors are from Bosch Sensortec, and an important consideration for sensor selection was low power dissipation. Figure 2 shows the WSN used to collect and transmit the data wirelessly. The sampling rate for the accelerometer and the gyroscope was 10 samples per second. Each data point contained acceleration in three axes, angular velocity in three axes, a timestamp, and a counter value. The timestamp and the counter value are used to check the validity of the data.

### Sensor attachment and camera setup

**Figure 3.**
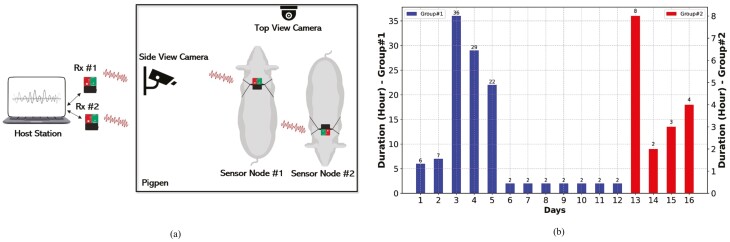
System setup (a). The video data were collected using camera for labeling purposes. The motion data were transmitted wirelessly from sensor node to host station; the duration of data collection—Group #1 (blue) is from two large pigs and Group #2 (red) is from two small pigs (b).

The sensor node was placed inside a plastic box and then attached to the pig’s back using a harness. There was a space between the harness and the pigskin so that the pig would not be annoyed and behave naturally. No metal was used for the pig’s safety. One camera was mounted on the ceiling and the other at the side of the pigpen. Both cameras recorded videos at a speed of 30 frames per second. The video recordings provided ground truth for data annotations. Figure 3a illustrates the attachments of the sensor nodes and cameras.

### Data collection

A major objective of our data collection was to build a data set representing the diverse natural behaviors of pigs. We collected data from four pigs of different sizes that were 5 wk postweaning. We grouped the four pigs into two groups based on their sizes. Group #1 consisted of the two larger pigs and Group #2 the two smaller pigs. The pigpen was large enough for the pigs to move around freely during the data collection. Meanwhile, sufficient food and water were provided in the pigpen so that they could eat and drink at any time. A few toys were provided for the pigs as well.

One hundred and thirty-one hours of data were collected for 16 d over the span of 2 mo. One evaluator monitored the pigs’ behaviors using a closed-circuit television in a nearby room and checked in the event a pig attempted to damage the sensor node. The data were collected at two different periods for a day, which helped diversify the collected data. Each period was adjusted accordingly to the availability of the farm manager.

The period of data collection is summarized in Figure 3b. For the large pigs in Group #1, 114 h of data were collected for 12 d across approximately 6 wk. The longest period was 36 h on day 3, which means the data were continuously collected for 36 h from two pigs. For the smaller pigs in Group #2, 17 h of data were collected for 4 d across about 2 wk.

#### Observed behaviors and labeling

We reviewed 13 h of video recordings after the first 2 d of data collection and classified pigs’ behaviors into seven different behaviors listed in Table 1. “Eating” and “lying” activities were the majority behaviors with 83%, while “drinking” and “sitting” only accounted for 0.73%. When a pig was out of sight, it was labeled as “unknown.” We excluded less frequent behaviors, including “sitting” and “drinking” as well as “unknown” from the analysis.

**Table 1.** The composition of pig behaviors in the collected data

Behavior	Description	Percentage (%)
Eating	Consuming food actively	49.40
Lying	Lying posture in idle	33.33
Walking	Moving on the ground with head up	7.87
Standing	The animal is standing and inactive	1.76
Playing	Sniffing, chewing, or moving the pig toy	0.48
Drinking	Drinking water	0.49
Sitting	The front legs are up, while the back legs are down	0.24
Unknown	The pig is out of the vision	6.43
Total		100

Three personnel performed the labeling initially, three more personnel reviewed and verified the labels, and one person finalized the labels. We used Data Capture Lab software, the community edition by SensiMl, to annotate the data (https://sensiml.com/products/data-capture-lab/) (SensiML, 2021).

#### Data preprocessing

Data preprocessing affects the accuracy of a machine learning model (Deshmukh et al., 2015; Huang et al., 2015). The collected data set was preprocessed through detection and removal of outliers, interpolation, and standardization.

One hundred and thirty-one hours of data were collected for 16 d. As accelerometers and gyroscopes often contain anomalies due to a variety of causes, such as sensor failure, depleted WSN battery, and transmission error. Detection and removal of outliers are necessary to improve the performance of machine learning algorithms (Ester et al., 1996; Syafrudin et al., 2018). We adopted a method based on the interquartile range (Acuna and Rodriguez, 2004; Sadik and Gruenwald, 2011; Yin et al., 2016). An accelerometer data point was considered an outlier if it was outside of the upper/lower fence and beyond the accelerometer range (=16 × 9.81 m/s^2^). The same rule was applied to the gyroscope data points, in which the range was ±2,000 °/s. The missing values of the data due to the removal of outliers were replaced by **NaN** (not a number) values. We adopted the linear interpolation method to fill in NaN values.

We collected data from four pigs over 8 wk, and the measurement conditions changed over time. For example, as a pig grows, the behavior pattern of the pig changes. To minimize deviations in each pig’s data over time and dependency on individual pigs, we normalized each pig’s data to range from 0 to 1 and integrated individual pigs’ data into one data set for segmentation and analysis.

### Segmentation

Sliding and adaptive windows are commonly used techniques for segmentation (Pirttikangas et al., 2006; Munguia Tapia, 2008). We adopted the sliding and adaptive window methods for data segmentation and compared the performance of five machine learning algorithms.

#### Sliding window

**Figure 4.**
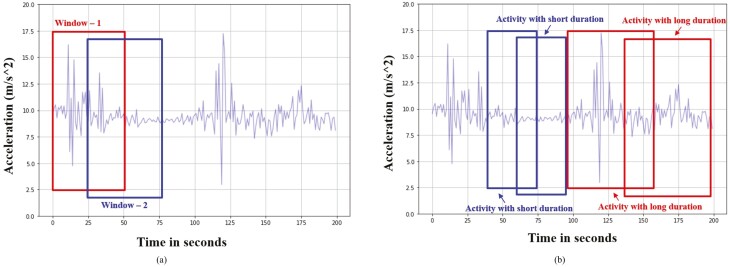
Sliding windows of the window size of 50 s and the step size of 25 s. All windows have the same window size (a); adaptive windows. The blue windows of 30 s and the red windows of 50 s were designed for short and long activities, respectively (b).

Signals for the sliding windows were split into windows of a fixed size. The sliding windows are suitable for classifying static behaviors, such as “walking,” “eating,” and periodic behaviors, such as “lying” (Banos et al., 2014). Figure 4a illustrates the first two windows for the sliding window method. The signals are the magnitude vector of the acceleration data in the x-, y-, and z-axes. The WS determines the size of segmented acceleration data, whereas the SS defines the distance between two consecutive windows or the size of increment when segmenting the acceleration data. With WS 50 and SS 25, the first window starts at 0 and ends at 50, and the second window starts at 25 and ends at 75. By adjusting the SS, the two consecutive windows can overlap to increase the resolution of the segments and capture the transitions between activities (Munguia Tapia, 2008; Banos et al., 2014). On the one hand, the sliding window method has relatively low computational complexity because it treats all behaviors with the same WS and SS. On the other hand, the fixed WS lacks flexibility and introduces noisy features by including multiple short activities, resulting in low classification accuracy (Gu et al., 2009).

#### Adaptive window

Adaptive windows are variations of sliding windows. Instead of experimenting with the uniform WS and SS, it finds the precise duration by searching the optimal WS and SS for different activities. Figure 4b illustrates the adaptive windows, in which the blue window has the size of 30 s, aiming for short activities, whereas the red window is 50 s for long activities.

### Feature extraction

Discriminative features can differentiate animal behaviors. For instance, the standard deviation can distinguish “walking” from “standing,” and the Fast Fourier transform coefficients can help differentiate between “walking” and “running” (Oniga and Sütő, 2014). We calculated 224 features in total from both time and frequency domains. All such features were extracted from the three axes of the accelerometer and the gyroscope in time and frequency domains. These features included mean, standard deviation, maximum, minimum, interquartile range, max–min difference, positive and negative count, skewness, kurtosis, Euclidean norm, zero-crossing rate, the sum of square errors, the absolute sum of changes, and accelerometer pitch roll. This last feature, accelerometer pitch roll, is unique for accelerometers and intuitively indicates pigs’ activity (Plotz et al., 2011). The pitch (roll) is the angle between the x-axis (y-axis) acceleration and the projection of the point to the XZ (YZ) plane.

### Evaluation metrics and algorithms considered

To evaluate the performance of classification algorithms, we split the data set into two subsets using stratified cross-validation over the entire data set: 70% of the data set for training and 30% for testing sets. In stratified cross-validation, each class consisted of stratified random samples and split them into training and testing sets. After stratified cross-validation, the results could reflect the distribution of the original data set (Refaeilzadeh et al., 2009; Igareta, 2021).

To evaluate the performance of the classifiers, we used the precision, recall, and F1 scores, derived from the confusion matrix.

#### Precision

Precision indicates the fraction of the correctly classified positive instances among the predicted positive instances. It is related to type I error that the model believes the readings belong to an activity, but they actually do not. Precision was calculated as:


$$\begin{array}{l} Precision=\frac{TP}{TP+FP} \end{array}$$

where TP stands for true positive, which indicates the number of positive examples classified accurately. FP is false positive, which is the number of negative examples classified as positive (Kulkarni et al., 2020).

#### Recall

Recall is the fraction of the correctly classified positive instances among the positive instances. It is related to type II error that the model believes the readings do not belong to an activity, but they actually do. Recall was calculated as:


$$\begin{array}{l} Recall=\frac{TP}{TP+FN} \end{array}$$

where FN is false negative, which is the number of positive examples classified as negative.

#### F1 score

The F1 score reflects the combined performance of the precision and the recall. The F1 describes the harmonic mean of the precision and is calculated as:


$$\begin{array}{l} F1=2\times \frac{Recall\times Precision}{Recall+Precision} \end{array}$$

### Machine learning algorithms

A major goal of this research was to identify pigs’ behaviors using an accelerometer and a gyroscope. We selected five classification algorithms, specifically SVM, KNN, decision trees (**DT**), naive Bayes (**NB**), and random forest (**RF**). These models were implemented using the Scikit-Learn library.

#### Support vector machine

SVM is a binary classification method aiming to separate two classes with the maximum distance between them. We used the one vs. all method, which treats one behavior as positive and the rest as negative when dealing with multiple behaviors. The prediction was based on the highest confidence score. We used a linear kernel in SVM.

#### *K*-nearest neighbors

KNN is a nonparametric classification method that classifies the input based on the similarity of existing data. We chose KNN as it predicts the results without strong assumptions about the data set. Also, the variables do not need to be independent and identically distributed or linearly separable. The number of nearest neighbors *K* was set as eight neighbors.

#### Decision trees

DT is a nonparametric classification method that classifies the given input with learned decision rules, such as the Gini impurity we used. The decision surface could be viewed as a piecewise function through a combination of if-then-else statements. We did not limit the depth of the tree.

#### Naive Bayes

NB is a probabilistic classifier based on Bayes’ theorem with the assumption that the input features are conditionally independent. The model outputs the likelihood based on the input feature values by learning the class conditional probability density.

#### Random forest

RF is an ensemble learning method that trains multiple DT, and the results are based on the average probabilistic prediction. Each tree is trained from bootstrap samples of the original data set. The objective is to maintain the strength while minimizing the correlation. Therefore, the trees grow independently and aims to avoid making the same mistakes. RF was implemented using 100 ensembles.

## Results and Discussion

### Performance under sliding windows

**Figure 5.**
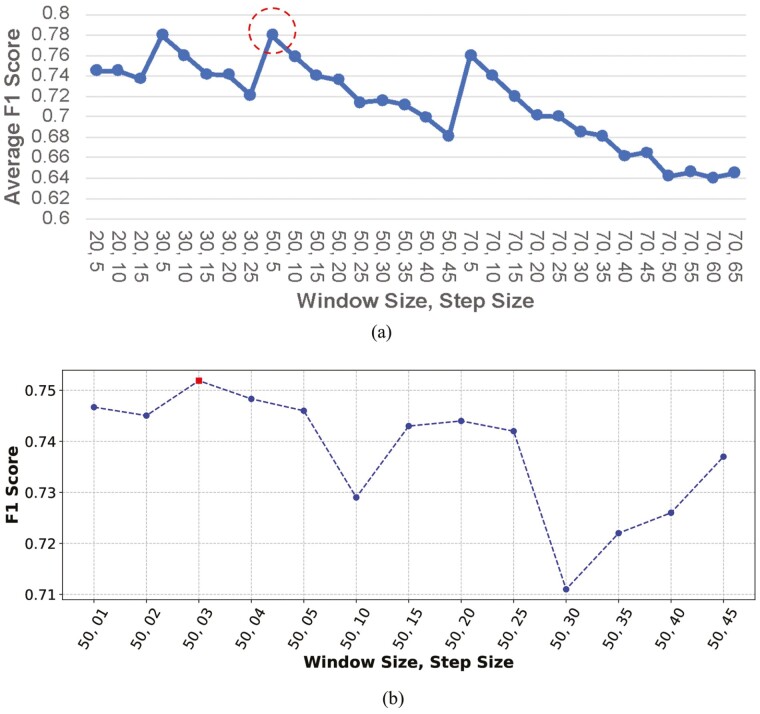
Average F1 score over five algorithms using the fixed sliding windows. The window and step sizes are represented in terms of the number of samples. Window size (WS) ranging from 20 samples (2 s) to 70 samples (7 s). Step size (SS) ranging from 5 samples (0.5 s) to 75 samples (7.5 s) (a). The best F1 score was obtained with WS 50 samples (5 s) and SS 3 samples (0.3 s) (b).

We measured the performances of the algorithms with the WS of 2, 3, 5, and 7 s and SS from 0.5 to 6.5 s with a 0.5-s increment (Figure 5a). Note that the sampling rate of the proposed WSN was 10 samples per second. The F1 scores were highest with the (WS 3 s, SS 0.5 s) and the (WS 5 s, SS 0.3 s) pairs. We chose the (WS 5 s, SS 0.3 s) pair because WS 5 s contains more samples than WS 3 s, so the calculated features could be stabler, and the models trained on those features are more robust. With WS 3 s, a 0.5-s spike in the motion data could inflate the mean and other values, thus confusing the models. Figure 5b shows the average F1 score of the five algorithms under the WS 5 s with varying SS. In general, the average performance decreased as SS increased. The highest average F1 score of 0.756 was achieved for the WS of 5 s (50 samples) and the SS of 0.3 s (3 samples).

Table 2 compared the average F1 score of the five algorithms for four different behaviors under the WS of 5.0 s and the SS of 0.3 s. RF and SVM achieved the highest F1 score for almost all behaviors. KNN achieved comparable results except for the two minority classes, “walking” and “standing.”

**Table 2.** Average F1 score under window size = 5 s and step size = 0.3 s for the sliding window method

Behaviors	DT	KNN	NB	RF	SVM^1^
Eating	0.95	0.96	0.91	0.97	0.97
Lying	0.95	0.96	0.91	0.96	0.96
Walking	0.78	0.82	0.78	0.84	0.87
Standing	0.28	0.15	0.21	0.39	0.39
Average	0.74	0.72	0.70	0.79	0.79

^1^Five classification algorithms applied: DT, decision trees; KNN, *k*-nearest neighbors; NB, naive Bayes; RF, random forests; SVM, support vector machine.

In summary, the sliding windows have a fixed WS and are incapable of fitting activities with different periods. As shown in Table 2, the sliding windows perform well for the major classes, “eating” and “lying,” but not for the minority classes “walking” and “standing.”

### Performance under adaptive windows

**Figure 6.**
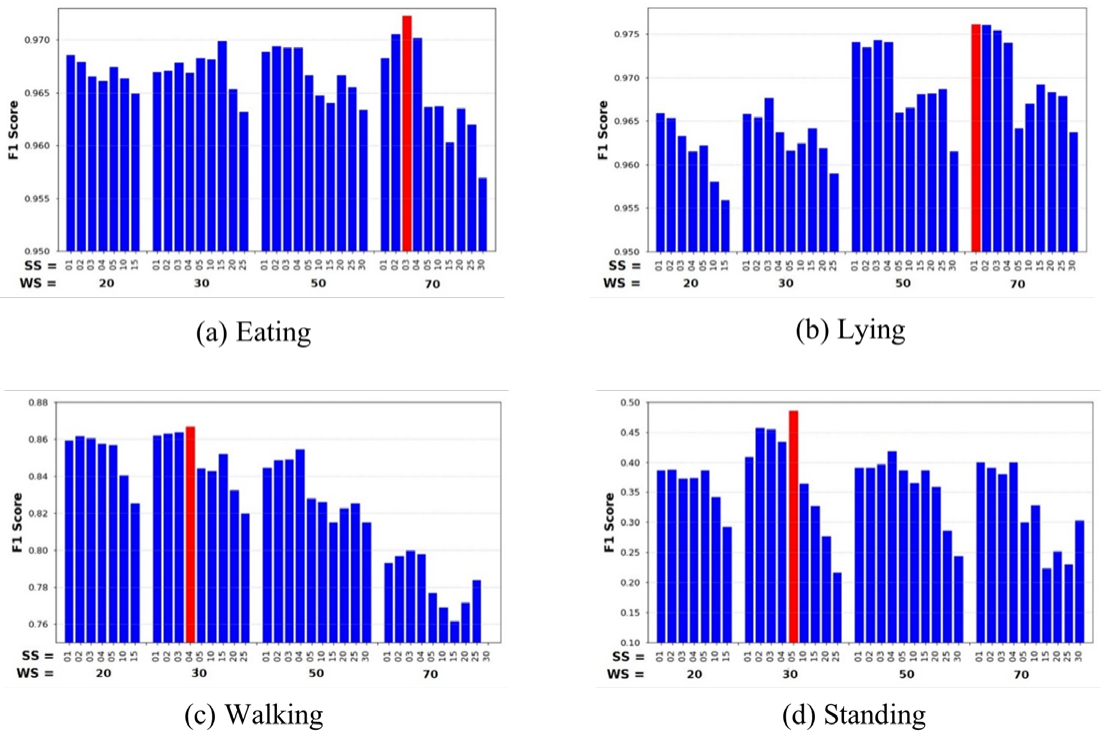
F1 Score versus Window Size and Step Size for RF Classifier. Bars of a figure are grouped under the same WS. The red bar of each figure indicates the F1 score under the optimal WS and MS. Note that WS and SS values are the numbers of samples, and the sampling rate is 10 samples per second. For example, WS = 20 samples (=2 s) and SS = 1 samples (=0.1 s). The figures indicate that the optimal window size is larger (smaller) for an activity with a longer (shorter) duration.

Instead of using the same WS and SS for all classes, we explored the optimal WS and SS using the grid search. The minority classes, “walking” and “standing” showed shorter durations than the major classes, “eating” and “lying,” so minority classes would perform better with shorter WS. Figure 6 shows the F1 score of the RF algorithm under various WS and SS. Two classes, “eating” and “lying,” achieved the highest performance under the WS of 7 s (70 samples), while “walking” and “standing” under the WS of 3 s (30 samples). Figure 6 verifies that the optimal WS of an activity class depends on the duration of the activity. By segmenting the input data with the customized WS, we can address the shortcoming of the sliding windows, specifically low performance for the minority classes “walking” and “standing.” The red bar in each class indicates the highest F1 score under the optimal WS and SS. The optimal WS and SS for the RF algorithm were 7 and 0.3 s for “eating,” 7 and 0.1 s for “lying,” 3 and 0.4 s for “walking,” and 3 and 0.5 s for “standing,” respectively. We used these adaptive windows to segment each activity to better capture the behavioral characteristics.

The adaptive windows performed better for the remaining machine learning algorithms considered in addition to RF. Table 3 compared the F1 scores of all algorithms with optimal WS and SS for each algorithm and behavior. The F1 scores increased for all algorithms and all behaviors, especially for the minority class “walking.” The trend was the same for the DT algorithm, which confirms that the adaptive windows are better for identifying pigs’ behaviors. The use of different WS and SS has been reported in human activity recognition (Banos et al., 2014; Li et al., 2018). By using different WS and SS for human activities of various duration, the model’s performance improved. Their results corroborated our findings in pig activity recognition. We found that the adaptive windows significantly improved the F1 scores for activities with varying durations. For example, the F1 score of “walking” for RF increased to 0.93 from 0.84, and the F1 score of “standing” increased to 0.91 from 0.39 under the sliding windows (Table 2). The RF algorithm achieved the highest F1 scores among all the five algorithms.

**Table 3.** F1 Scores under optimal window size and step size for each classification algorithm and each activity behavior

Behaviors	DT	KNN	NB	RF	SVM
Eating	0.95 (7.0, 3.0)^1^	0.88 (5.0, 1.0)	0.88 (5.0, 1.5)	0.98 (7.0, 0.3)	0.93 (7.0, 0.5)
Lying	0.97 (7.0, 2.5)	0.92 (5.0, 2.0)	0.87 (5.0, 3.5)	0.99 (7.0, 0.1)	0.92 (7.0, 3.5)
Walking	0.93 (3.0, 1.5)	0.87 (3.0, 0.5)	0.76 (2.0, 1.5)	0.93 (3.0, 0.3)	0.89 (3.0, 0.5)
Standing	0.90 (3.0, 1.0)	0.55 (2.0, 3.0)	0.52 (3.0, 3.5)	0.91 (3.0, 0.5)	0.80 (5.0, 0.5)
Average F1 score	0.94	0.81	0.76	0.95	0.88

^1^Each entry has three values: F1 score, window size and step size in seconds. Five classification algorithms applied: DT, decision trees; KNN, *k*-nearest neighbors; NB, naive Bayes; RF, random forests; SVM, support vector machine.

Tables 4 and 5 show the precision and recall values, respectively. As expected, those values also increased when the adaptive windows were used, resulting in an increase in F1 scores. In conclusion, the adaptive windows performed better than the sliding windows for classifications of pigs’ behaviors, especially for minority activities.

**Table 4.** Precision values under optimal window size and step size for each classification algorithm and each activity behavior

Behaviors	DT	KNN	NB	RF	SVM
Eating	0.95 (7.0, 3.0)^1^	0.81 (5.0, 1.0)	0.92 (5.0, 1.5)	0.99 (7.0, 0.3)	0.92 (7.0, 0.5)
Lying	0.96 (7.0, 2.5)	0.89 (5.0, 2.0)	0.87 (5.0, 3.5)	0.99 (7.0, 0.1)	0.93 (7.0, 3.5)
Walking	0.90 (3.0, 1.5)	0.92 (3.0, 0.5)	0.71 (2.0, 1.5)	0.90 (3.0, 0.3)	0.90 (3.0, 0.5)
Standing	0.91 (3.0, 1.0)	0.77 (2.0, 3.0)	0.45 (3.0, 3.5)	0.92 (3.0, 0.5)	0.81 (5.0, 0.5)
Average precision	0.93	0.85	0.74	0.95	0.89

^1^Each entry has three values: precision, window size and step size in seconds. Five classification algorithms applied: DT, decision trees; KNN, *k*-nearest neighbors; NB, naive Bayes; RF, random forests; SVM, support vector machine.

**Table 5.** Recall values under optimal window size and step size for each classification algorithm and each activity behavior

Behaviors	DT	KNN	NB	RF	SVM
Eating	0.95 (7.0, 3.0)^1^	0.96 (5.0, 1.0)	0.85 (5.0, 1.5)	0.96 (7.0, 0.3)	0.95 (7.0, 0.5)
Lying	0.98 (7.0, 2.5)	0.95 (5.0, 2.0)	0.87 (5.0, 3.5)	0.99 (7.0, 0.1)	0.91 (7.0, 3.5)
Walking	0.97 (3.0, 1.5)	0.82 (3.0, 0.5)	0.83 (2.0, 1.5)	0.97 (3.0, 0.3)	0.89 (3.0, 0.5)
Standing	0.90 (3.0, 1.0)	0.43 (2.0, 3.0)	0.63 (3.0, 3.5)	0.91 (3.0, 0.5)	0.79 (5.0, 0.5)
Average recall	0.95	0.79	0.80	0.96	0.88

^1^Each entry has three values: recall, window size and step size in seconds. Five classification algorithms applied: DT, decision trees; KNN, *k*-nearest neighbors; NB, naive Bayes; RF, random forests; SVM, support vector machine.

### Feature selection

We extracted 244 features in total from the acceleration and angular velocity in three axes. Some of the features were eliminated to decrease training time because they were redundant or insignificant. We adopted a recursive method using the RF algorithm. Each feature was scored after an iteration, and a certain percentage of the bottom features were eliminated in the cycle. We selected the top 25% of the features through the process by eliminating 183 features.

## Conclusions

This paper presents the behavior analysis using optimized WS and SS. We collected 131 h of acceleration and angular velocity data from pigs. We compared the model performance using two segmentation methods: the sliding and adaptive windows. To find the optimum adaptive WS, we explored different WS for individual activities. We showed that the models with adaptive windows achieved higher performance than the sliding windows, especially for activities with shorter durations. Compared with the DT, KNN, NB, and SVM, the RF algorithm with the adaptive windows achieved the highest accuracy.

Our future studies will include other behaviors, such as “sleeping” and “drinking,” which can also inform a pig’s health status. We will also consider using additional sensors to provide more comprehensive health assessments. To improve the classification performance further, we will explore other machine learning algorithms and advanced deep learning methods for pig behavior monitoring.

## Abbreviations

DT: decision trees
KNN: *k*-nearest neighbors
NaN: not a number
NB: naive Bayes
RF: random forest
SS: step size
SVM: support vector machine
WS: window size
WSN: wireless sensor node
